# Supplementary material for: Phase-Optimized Peristaltic Pumping by Integrated Microfluidic Logic
Source: Micromachines (Basel). 2022 Oct 20;13(10):1784. doi: 10.3390/mi13101784 (PMC9610095; doi:10.3390/mi13101784)
Supplement: Supplementary file 1 [file micromachines-13-01784-s001.zip › micromachines-1892928-supplementary.pdf]

Phase-Optimized Peristaltic Pumping by Integrated Microfluidic Logic

Supplementary Materials

Erik M. Werner, Benjamin Lam, and Elliot E. Hui

Contents:

Figure S1: Rapid prototyping process
Figure S2: Control of channel depth with a CO_2 laser
Figure S3: Working principle of an oscillator pump
Figure S4: Mixing performance quantification
Figure S5: Series vs parallel pump configuration
Figure S6: Valve deflection measurement
Figure S7: Experimental setup
Table S1: Iterative design progression

Video S1: Steady state oscillator pump droplet generation from 50 kPa constant vacuum source
Video S2: Electricity free droplet generation from 60 mL syringe pull
Video S3: Droplet generator response time

Calculation S1: Hydraulic resistance calculations
Calculation S2: Capillary number calculations

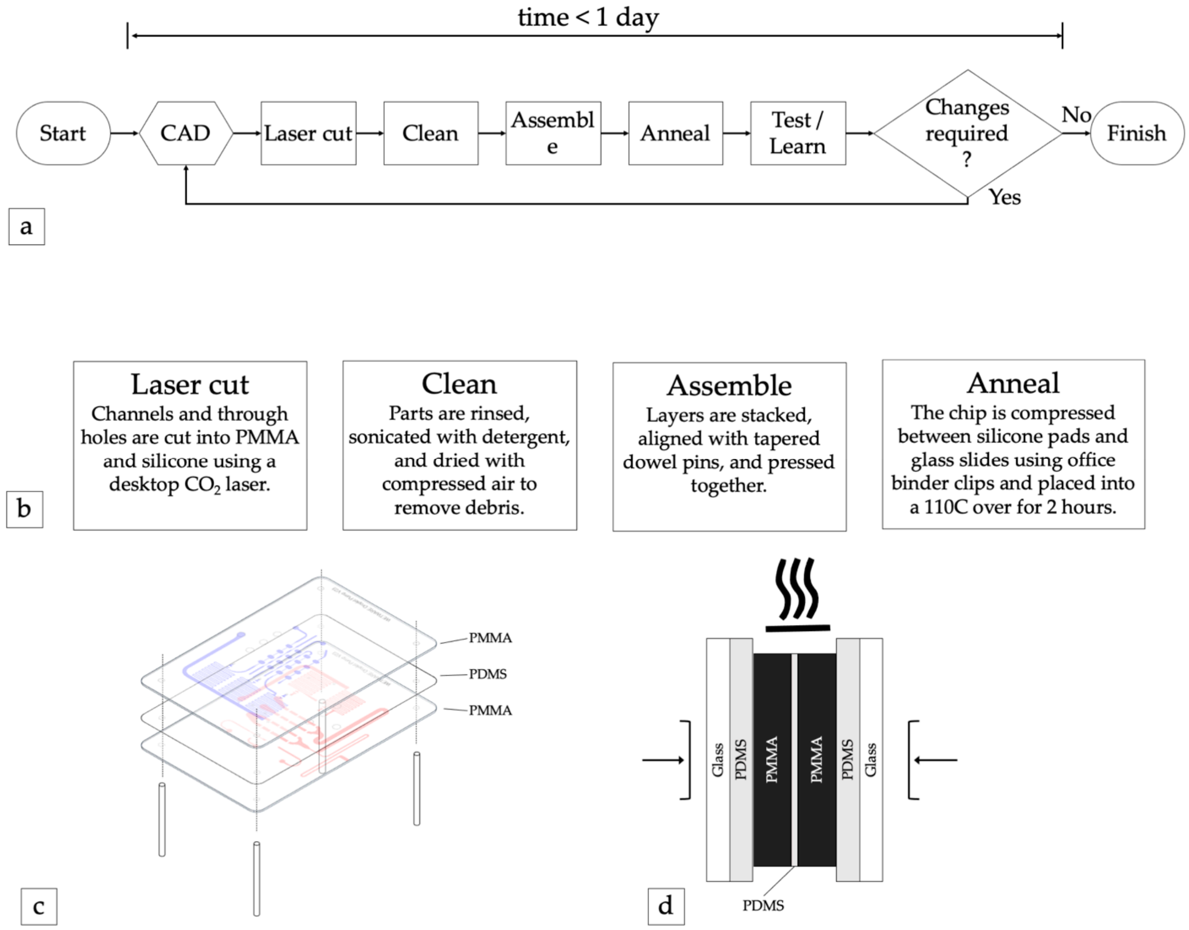

Figure S1: Rapid prototyping process for microfluidic digital logic circuits. **(a)** Design, build, test, learn process enabled through rapid prototyping **(b)** Fabrication method details **(c)** Alignment and assembly of device layers **(d)** Side view of assembled device during bonding. Devices were clamped between additional pieces of PDMS and glass slides for bonding.

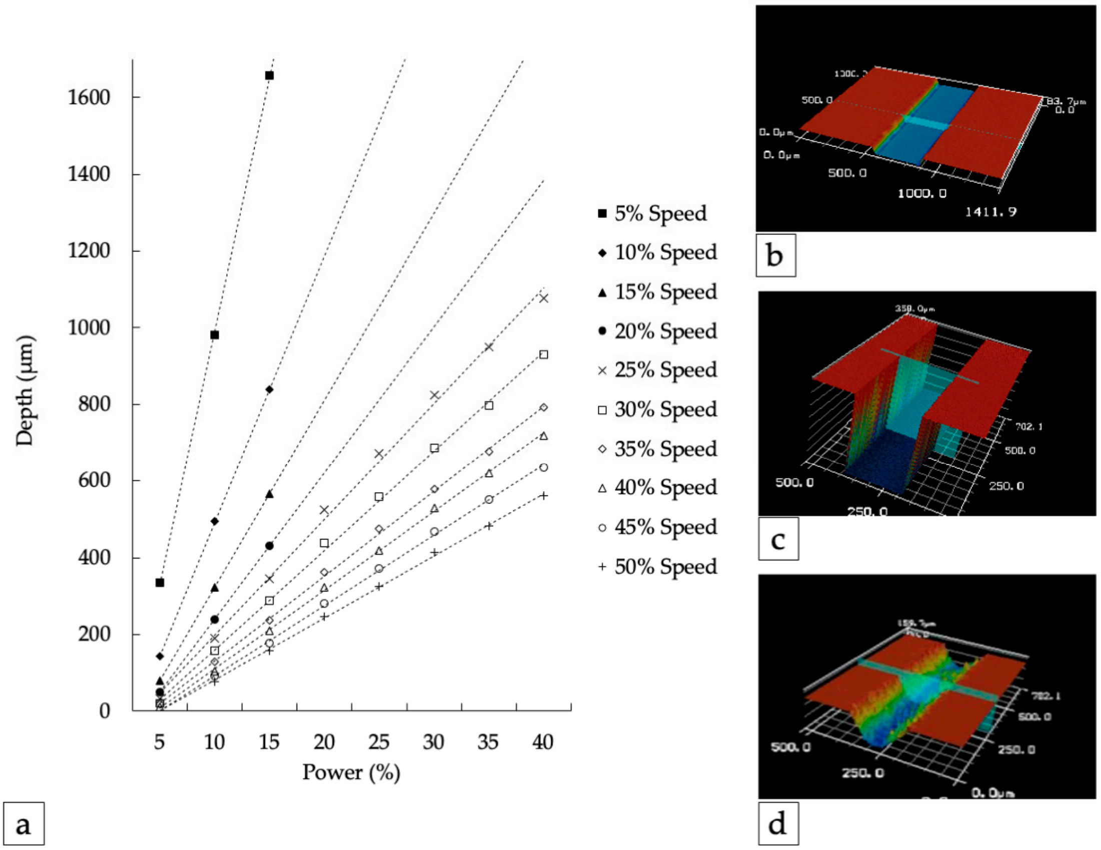

Figure S2: Depth control CO_2 of laser-fabricated microchannels. **(a)** Engraving depth vs laser power. Data are presented as the average depth at the center of the channel. Dotted lines represent linear fits. Speed values are presented as a percent of the maximum raster speed of the laser (40 in/sec). Power values are presented as a percent of the maximum power of the laser (60W). **(b-d)** Surface profiles of channels produced by wet etching (250 µm width x 83 µm height), CNC micro milling (200 µm x 350 µm), and CO_2 laser engraving (200 µm x 100 µm).

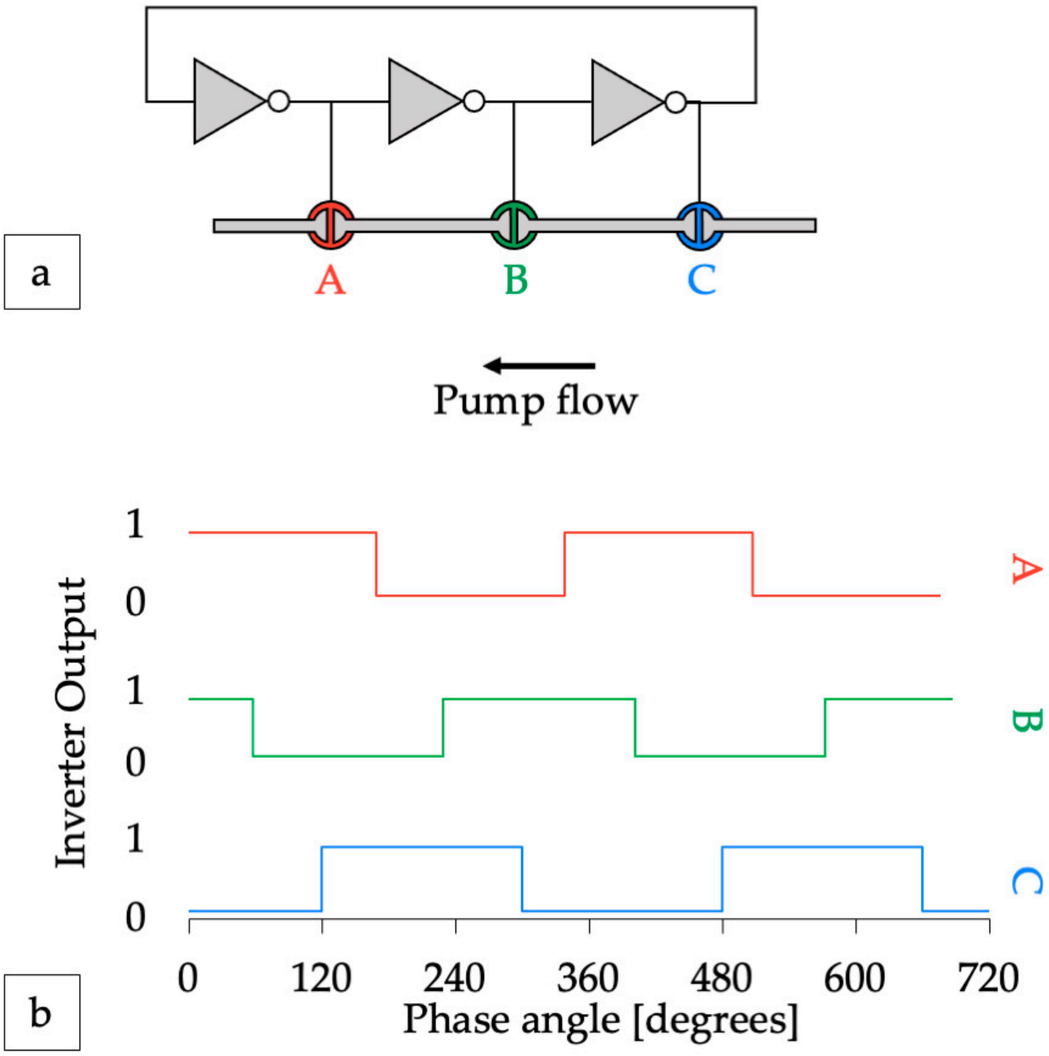

Figure S3: Oscillator pump working principle. **(a)** Each node of a three-inverter ring oscillator is connected to pump valves (A,B,C). **(b)** The ideal waveform output from the ring oscillator generates a pattern that produces peristaltic pumping.

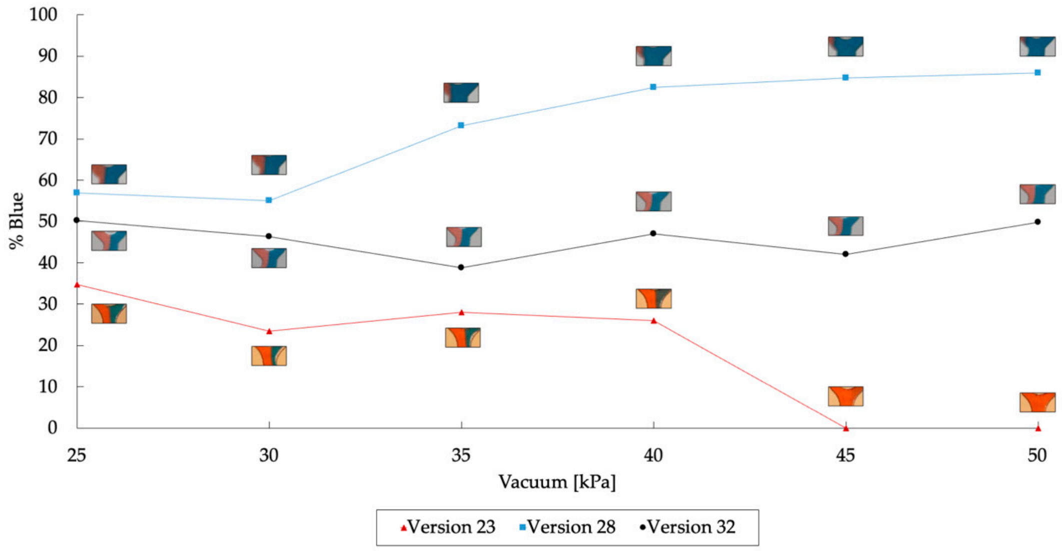

Figure S4: Mixing performance was quantified by measuring the distance of the red/blue interface from the center of the channel. When pumps are not synchronized, the flows become unbalanced when vacuum power is increased.

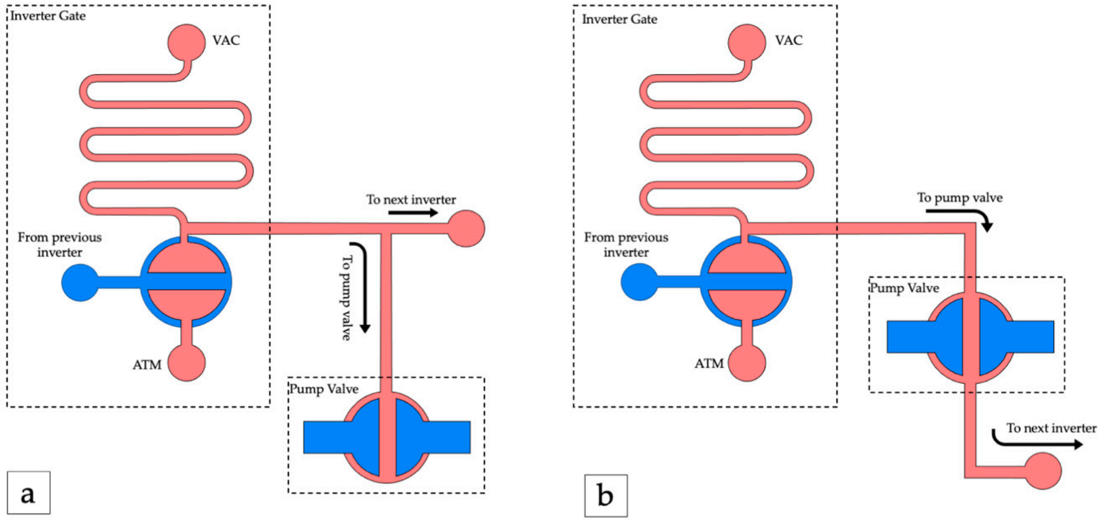

Figure S5: Parallel and series pump valve arrangements **(a)** Parallel circuit: The inverter output signal is split to connect both a pump valve and the next inverter **(b)** Series circuit: The inverter output signal is routed through the pump valve to connect to the next inverter.

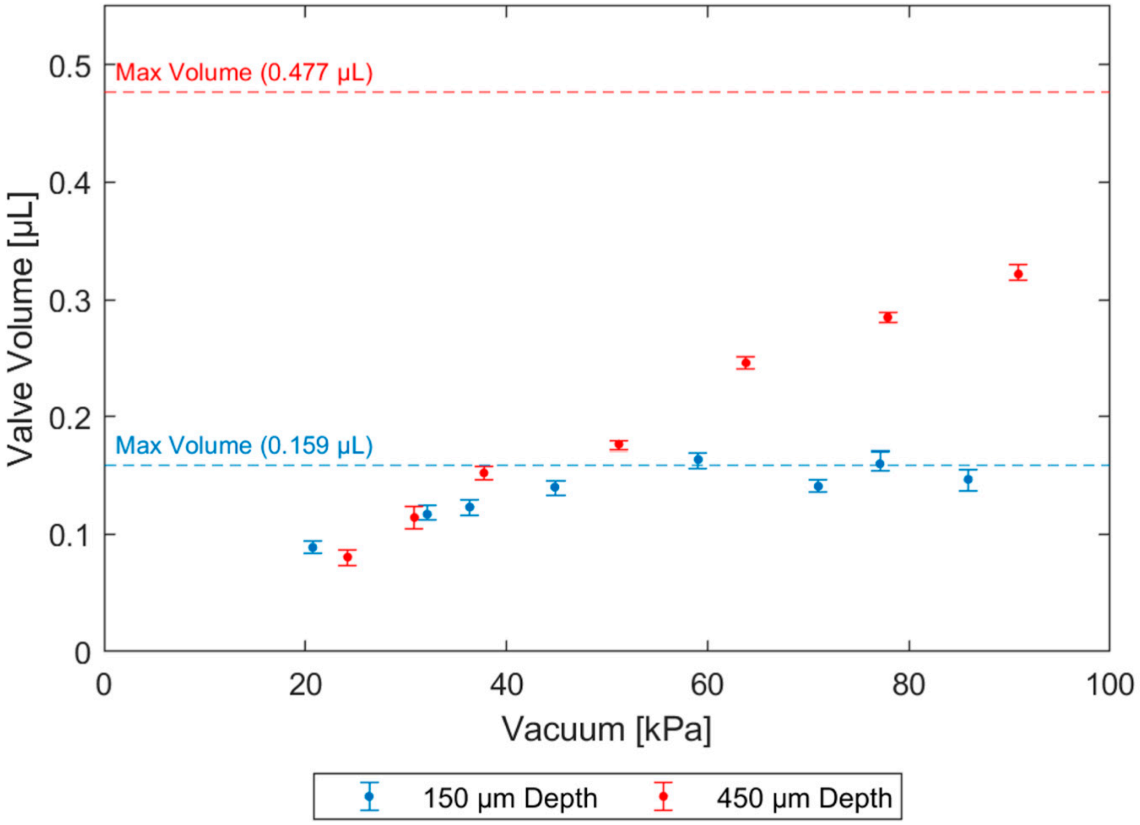

Figure S6: Valve volume measurement. The volume of water displaced by a single valve was measured for a shallow valve (150 µm, blue) and a deep valve (450 µm, red). The behavior of both valves is similar at low pressures. At higher pressures, shallow valves (blue) become limited by the depth of the displacement chamber and approach the maximum volume of the chamber at around 50 kPa, while deep valves (red) continue to displace more volume as actuation vacuum increases. The maximum volumes were calculated assuming the elastomeric membrane fully conforms to the walls of the valve.

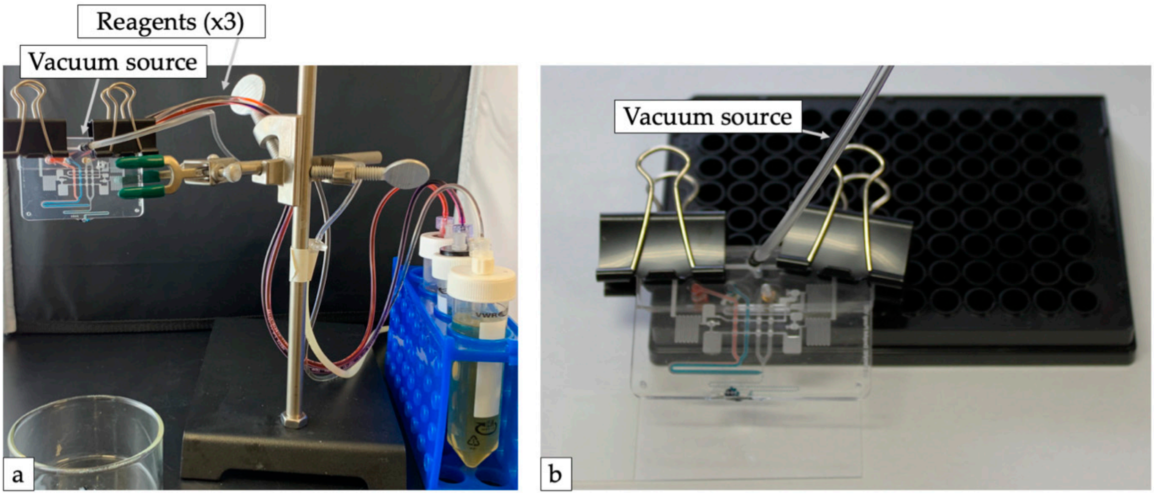

Figure S7: Photos of compact experimental setup **(a)** The droplet system requires only a vacuum source and connections to reagents. **(b)** Using short lengths of tubing, droplets are generated directly from the wells of a 96 well plate.

Ver	Drawing	Changes	Results	Hypothesis
2	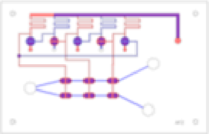	Initial design: One oscillator drives two pumps to mix liquids. This design produces drops of mixed liquid, not droplets.	Pumps produced unequal flow rates, with the pump farthest from the oscillator pumping too slowly.	Compressible flow causing pump valves further from the oscillator to displace less volume.
4	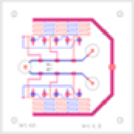	Added additional oscillator to balance pump flow rates. Reduced oscillator frequency and pump valve diameter to lower flow rate.	Even mixing was observed, but smaller drop volumes were wanted. A water-in-oil droplet design was investigated.	Adding an oil pump with a T-junction will produce water-in-oil droplets.
6	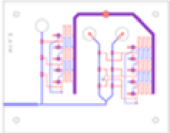	Added T-junction and additional oscillator pump to make water in oil droplets. Aqueous phase pumps were consolidated to use single oscillator design by equalizing pump valve path lengths.	Droplets were observed, but only when aqueous phase pump flow rate was reduced.	Increasing oil pump flow rate will produce droplets at higher aqueous phase flow rates.
8	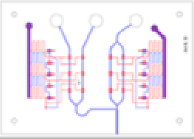	Added additional pump to oil phase to increase flow rate. Separated oil and aqueous pump vacuum supply lines to allow independent flow rate control.	Droplets were observed at most flow rates.	Independent flow rate control of aqueous phase and oil phase not necessary with additional oil pump.
14	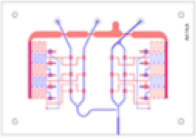	Connected oscillator power lines together to simplify control. Improved world-to-chip connection by using smaller fittings. Decreased T-junction nozzle width to produce smaller droplets.	Droplet size and uniformity varied if oil and aqueous pumps were not synchronized.	Oil and aqueous pumps must be synchronized to produce uniform droplets.
18	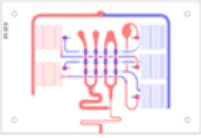	Synchronized pump stages to a single oscillator circuit to improve droplet uniformity. Connected all pump valves except the final pump row in series with the oscillator.	Droplet volume varied with total flow rate. Did not self-prime reliably.	Adding capacitors, check valves, or additional inverter stages will improve pump synchronization.
27	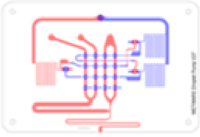	Reduced oscillator to three inverters to reduce the number of forward flow pulses per cycle from 3 to 2 and decrease total flow rate. Connected final pump row in series with the oscillator. Added check valves to all pump outputs.	Weak aqueous phase pumps. The right aqueous phase pump overpowered the left pump at higher input pressures.	Compressible flow still causing delays between pumps in parallel. Slowing pump waveform will improve valve synchronization and pump uniformity.
32	3 mm 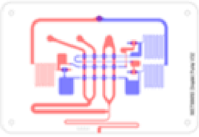	Removed check valves. Adjusted valve opening and closing times with large capacitors in series before and after all pump stages. Reduced depth of pump valve displacement chambers. Moved pneumatic and liquid connectors to opposite sides to simplify operation.	All pumps prime evenly, uniform-sized droplets produced at all flow rates.	-

Table S1: A selection of design revisions illustrate the progression of the optimization process. Channels on the top and bottom layers of the chip are shown in blue and red, respectively. The results of each design revision were used to drive incremental changes in the next revision. Scale Bar = 3 mm.

Video S1: Steady state droplet generation from 50 kPa constant vacuum source. The oscillator pump droplet system is connected to an electric vacuum pump via a vacuum regulator set to 50 kPa. 21 μL droplets are produced at a rate of 2.2 Hz with a 1.1% CV. The video is presented in real time.

Video S2: Electricity free droplet generation. A 60 mL locking syringe is directly connected to the oscillator pump droplet system. The plunger is pulled and locked at the 60 mL mark, generating approximately 90 kPa of vacuum pressure that gradually decreases as the droplet pump operates. The system runs for over 14 minutes from this vacuum supply, producing 465 droplets. The video is presented in real time.

Video S3: Oscillator pump droplet generator response time. A 70 kPa constant vacuum source is connected to the oscillator pump droplet system and switched on and off via an external solenoid valve. The system responds almost instantly and resumes steady state droplet generation after expelling liquid remaining in the T-junction from the previous operation. The video is presented in real time.

Calculation S1: Hydraulic Resistance
The hydraulic resistance of a rectangular and square microchannels were calculated using the equations S1 and S2.[37]

$$R_{h,rect} \cong \frac{12\,\mu\,L}{(w\,h^3)(1-((0.630\,h)} \qquad \text{(S1)}$$

$$R_{h,square} \cong \frac{28.4\,\mu\,L}{h^4} \qquad \text{(S2)}$$

Where L, w, h are the channel dimensions and $\mu = 18.13\ \mu Pa * s$, dynamic viscosity of air at 20°C.[38]

Resistor channels: (200 µm x 100 µm): $R_h \cong 1.6 \frac{g}{mm^5 s}$ per mm
Interconnect channels: (400 µm x 400 µm): $R_h \cong 2.0 * 10^{-2} \frac{g}{mm^5 s}$ per mm
Power bus channels: (1000 µm x 1000 µm): $R_h \cong 5.1 * 10^{-4} \frac{g}{mm^5 s}$ per mm

Calculation S2: Capillary Number
The capillary number for the continuous phase was calculated using equation S3[39]

$$Ca = \frac{\mu_c v_c}{\gamma} \qquad \text{(S3)}$$

where $\mu_c = 33\ mPa * s$ is the dynamic viscosity of the continuous phase,[40] v_c is the mean speed of the continuous as phase extracted from video, and $\gamma = 24.8\ \frac{dyn}{cm}$ is the interfacial tension between the continuous and disperse phases.[41]
